# Role of Btg2 in the Progression of a PDGF-Induced Oligodendroglioma Model

**DOI:** 10.3390/ijms131114667

**Published:** 2012-11-12

**Authors:** Irene Appolloni, Sebastiano Curreli, Sara Caviglia, Manuela Barilari, Eleonora Gambini, Aldo Pagano, Paolo Malatesta

**Affiliations:** 1 IRCCS-AOU San Martino-IST, Largo Rosanna Benzi 10, 16132 Genoa, Italy; E-Mails: irene.appolloni@istge.it (I.A.); manuelabarilari@hotmail.com (M.B.); aldo.pagano@unige.it (A.P.); 2 Department of Experimental Medicine (DiMES), University of Genoa, 16132 Genoa, Italy; E-Mails: sebastiano.curreli@iit.it (S.C.); sara.caviglia@imls.uzh.ch (S.C.); eleonora.gambini@gmail.com (E.G.)

**Keywords:** glioma progression, tumor suppressor, high grade oligodendroglioma

## Abstract

Tumor progression is a key aspect in oncology. Not even the overexpression of a powerful oncogenic stimulus such as platelet derived growth factor-B (PDGF-B) is sufficient *per se* to confer full malignancy to cells. In previous studies we showed that neural progenitors overexpressing PDGF-B need to undergo progression to acquire the capability to give rise to secondary tumor following transplant. By comparing the expression profile of PDGF-expressing cells before and after progression, we found that progressed tumors consistently downregulate the expression of the antiproliferative gene Btg2. We therefore tested whether the downregulation of Btg2 is sufficient and necessary for glioma progression with loss and gain of function experiments. Our results show that downregulation of Btg2 is not sufficient but is necessary for tumor progression since the re-introduction of Btg2 in fully progressed tumors dramatically impairs their gliomagenic potential. These results suggest an important role of Btg2 in glioma progression. Accordingly with this view, the analysis of public datasets of human gliomas showed that reduced level of Btg2 expression correlates with a significantly worse prognosis.

## 1. Introduction

We recently developed a model of high grade glioma induced by transduction of PDGF-B in embryonic mouse brain. Although at the time of injection targeted cells are able to give rise to all the three main lineages of the adult brain, namely neurons, astrocytes and oligodendrocytes, all the tumors generated are oligodendrogliomas [1]. We also formally demonstrated that these tumors undergo progression from low to high grade, acquiring the capability to give rise to secondary tumors following intracranial transplantation in adult mice [2]. The model is ideal to study aspects of early phases of tumor progression that cannot be approached in patients, where tumors are normally detected only when they become symptomatic. Tumor progression is generally associated with the acquisition of additional molecular defects, mainly involving the loss of tumor suppressor molecules. Particularly relevant for glioma progression is the impairment of TP53 pathway [3,4], although not necessarily involving changes in the level of TP53 itself, but rather of its downstream targets [5]. We found that during the progression of PDGF-induced glioma model, the expression of TP53 is not grossly altered although one of its downstream target, Btg2, is consistently downregulated in all tumor tested. Btg2 is a member of a small family of antiproliferative genes, and is known to regulate the proliferation of neural progenitor cells during neurogenesis, promoting neurogenic asymmetric divisions [6,7]. The loss of Btg2 has been already implicated in cell transformation in different tumors, including prostate cancer [8], lung cancer [9] and breast cancer [10], and its expression has been shown to inhibit the formation of preneoplastic lesion in a model of medulloblastoma [11]. Here we examined the effects of Btg2 downregulation and overexpression on the tumorigenicity of PDGF-induced oligodendrogliomas.

## 2. Results and Discussion

To characterize the molecular events responsible for the progression from low to high grade oligodendrogliomas, we induced tumors by *in utero* transducing embryonic brains with a retroviral vector carrying PDGF-B and EGFP as reporter. All the transduced animals that survived after birth developed lethal hyperplasiae, classifiable in two distinct groups of latency. As previously described, hyperplasiae giving rise to neurological symptoms before 90 days showed typical characteristics of low grade tumors and we were not able to generate secondary tumors following transplant. In contrast, tumors with a late onset of symptoms showed typical feature of high grade glioma [2]. Both low and high grade tumors were dissociated and GFP-positive cells were separated with a fluorescence activated cell sorter. RNA extracted from low and high grade tumors was then extracted and analyzed with Affymetrix microarrays.

Since a first analysis showed that the expression of TP53 by itself was not significantly altered during progression, we concentrated our analysis on known downstream target of TP53 ([12]; Table 1), which modulation could better reveal an impairment of the pathway. This analysis showed that during glioma progression the majority of the direct targets of TP53 were almost unchanged, whereas the most significantly modulated was Btg2, which expression level was lower in high grade than in low grade gliomas.

**Figure 1:**
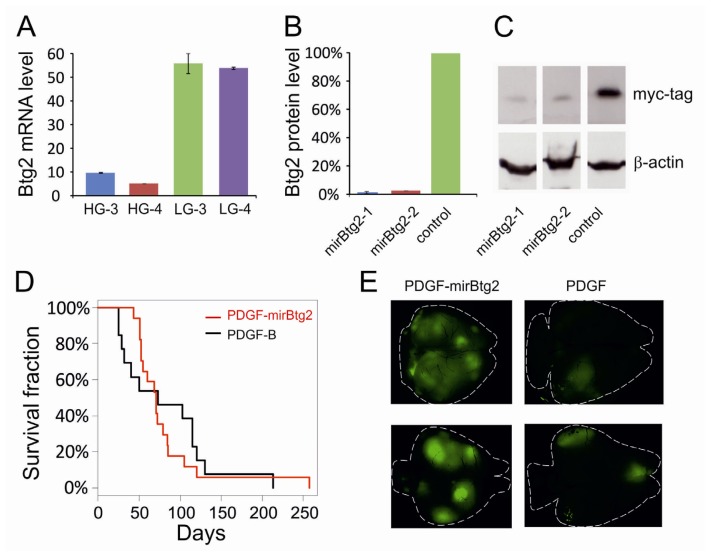
(**A**) The histogram shows the level of Btg2 mRNA in high grade (HG) and low grade (LG) PDGF-induced gliomas measured by quantitative PCR and normalized to the rpl41 housekeeping gene; (**B**–**C**) Quantification of the protein level of Btg2 in HEK293 cells cotransfected with myc-tagged Btg2 and vectors carrying artificial microRNAs against Btg2 or with a control vector (**B**) and corresponding western blot picture (**C**); (**D**) Kaplan-Meier plot of animals injected with PDGF-mirBtg2 or PDGF-B vectors at embryonic stage; (**E**) Fluorescence images of brains showing glioma masses induced by the embryonic infection with PDGF-mirBtg2 or PDGF-B retroviral vectors.

This difference of Btg2 levels in low and high grade tumors was further confirmed by real time RT-PCR in independently generated tumors (Figure 1A).

### 2.1. Downregulation of Btg2

To analyze the role of Btg2 in tumor progression, we asked whether its downregulation, together with PDGF-B overexpression, causes the formation of tumors with malignant traits from the very beginning. We therefore produced retroviral vectors co-expressing EmGFP, PDGF-B and an artificially designed microRNA targeting Btg2 (PDGF-mirBtg2). The efficacy of PDGF-mirBtg2 vector in silencing Btg2 was tested by cotransfecting it together with a myc-tagged Btg2 plasmid on HEK293. Western blot analysis showed that the engineered miRNA caused the reduction of Btg2 protein to about 3% of the level of cell cotransfected with a control vector (Figure 1B,C).

We then injected PDGF-mirBtg2 virus in the telencephalic vesicles of E14 mouse embryos and we monitored the survival of the animals after their birth. Kaplan-Meyer rank test analysis showed that the survival of the animals injected with PDGF-mirBtg2 was indistinguishable from that of control animals transduced with the vector expressing PDGF-B alone (Figure 1D). The appearance of tumors generated by PDGF-mirBtg2 was highly multifocal, showing more areas of GFP-labeled cells than tumors generated by PDGF-B alone (Figure 1E). To confirm this effect, we sampled the number of foci in each brain by analyzing pictures of the whole dorsal side of eight brains transduced with PDGF-mirBtg2 and eight brains transduced with PDGF-B alone with a partially automated system based on ImageJ. With this method we found in average 10 foci in the pictures of PDGF-mirBtg2 induced tumors and 3.6 in those of the control brains (*t* test *p* < 0.01; Figure S1). Induced tumors were further analysed by fixing, sectioning and staining them with different markers. PDGF-mirBtg2 induced gliomas were found immunopositive for Olig2 and Ng2 (Figure S2A,C) and negative for GFAP (Figure S2E), resulting indistinguishable from tumors induced by PDGF alone (Figure S2B,D,F; [1]).

Finally, in order to challenge their tumorigenic potential, 12 tumors induced by PDGF-mirBtg2 eliciting symptoms at different times (Table 2), were dissociated and transplanted in three adult animals. Similarly to what we previously found in tumors induced by PDGF-B alone, only tumors giving rise to symptoms after more than 90 days were able to generate secondary tumors [2], showing that the downregulation of Btg2 is not able, by itself, to speed up the tumor progression.

The increased number of hyperplasic foci induced by PDGF-mirBtg2 would be expected to increase the probability to develop high grade tumors. Consequently, it would be expected that the transplant of a given amount of EGFP-labeled cells derived from a PDGF-mirBtg2-induced tumor resulted in the generation of secondary tumors more easily (and earlier) than the transplant of the same amount of EGFP-labeled cells derived from a tumor induced by PDGF-B alone. This however is not the case, and our data show that PDGF-mirBtg2-induced tumors acquire the ability to generate secondary tumors after a progression that takes the same time as that of gliomas induced by PDGF-B alone. A possible explanation of this observation is that the increase of the number of hyperplasic foci automatically causes an increase of EGFP-labeled cells, including those lacking malignant features. The proportion between tumorigenic and non-tumorigenic EGFP-labeled cells is therefore likely constant. This may explain why we do not observe an increase of efficiency in generating secondary tumors when we transplant a given number of PDGF-mirBtg2 expressing cells.

### 2.2. Upregulation of Btg2

Having established that the downregulation of Btg2 by itself is not sufficient to confer fully malignant characteristics to PDGF-induced tumor, we asked whether it is necessary for the malignant phenotype. We therefore analyzed the effect of the re-introducion of Btg2 in fully progressed PDGF-induced glioma cells.

**Figure 2:**
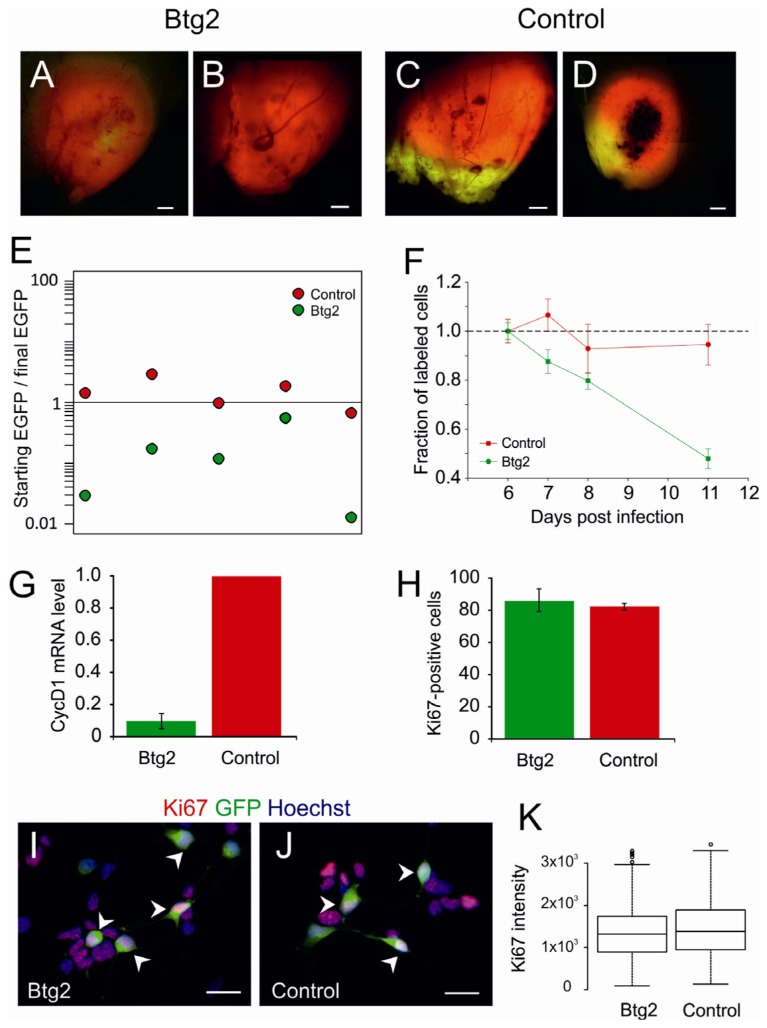
(**A**–**D**) Fluorescence images of tumors generated in the competition assay. All glioma cells are labeled in red since they derived from a PDGF/DsRed-induced tumor. Glioma cells additionally transduced with Btg2- or control-retroviral vector are expressing EGFP and are also labeled in green therefore appearing yellow due to the double labeling; (**E**) Ratio between starting and final percentage of double labeled cells in the competition assay; (**F**) Fraction of EGFP-positive glioma cells following transduction with Btg2 or control vector at subsequent passages; (**G**) CyclinD1 mRNA level in PDGF-induced gliomas 48 h after transduction with Btg2-expressing or control retroviral vectors measured by quantitative PCR; (**H**) Quantification of the percentage of Ki67-immunopositive cells in PDGF-induced glioma cells transduced with Btg2-expressing or control retroviral vectors; (**I**–**J**) Example of fluorescence microphotographs quantified in H showing in red Ki67 immunoreactivity, in green EGFP and in blue nuclei stained with Hoechst 33324. Arrowheads point to transduced cells immunopositive for Ki67; (**K**) Boxplot representing the quantification of the intensity of Ki67 in Btg2 or control transduced cells. Scale bars: 500 μm (**A**–**D**), 20 μm (**G**–**H**).

Fully progressed PDGF-induced oligodendrogliomas, labeled with DsRed, were dissociated and transduced with an additional retroviral vector encoding for Btg2 and EGFP (or EGFP only, as control) to obtain cultures containing between 20% and 50% of transduced cells. The cultures were then transplanted in adult mice, setting an *in vivo* competition assay [13]. Mice were killed as soon as they showed the onset of neurological symptoms (hyper-reactivity, imbalanced stance and gait, paralysis) and their brains were examined with a fluorescent microscope. All the tumors generated from the culture transduced with Btg2 appeared labeled with DsRed (Figure 2A,B) but only one of them showed EGFP positive areas. In contrast, tumors generated by the transplant of the control culture contained both DsRed-only and DsRed-EGFP double labeled areas (Figure 2C,D).

In order to quantify the percentage of double labeled cells, one brain injected with a mixed culture containing 50% of Btg2-transduced cells and one brain injected with a mixed culture containing 50% of EGFP-only-transduced control cells were fixed and sectioned. Sections were counterstained with Hoechst33342 and photographed with a fluorescent microscope (Figure S3A,B). An automated system based on ImageJ [14] was then used to count Ds-Red positive and Ds-Red/EGFP double positive cells in sample sections. The percentage of Btg2 transduced cells appeared strongly reduced in comparison to the percentage of EGFP-only transduced cells in the control (4% ± 1% *versus* 67% ± 11%, respectively). These percentages, however, were highly variable one section to another of the same brain, as shown in Figure S3C, due to non-homogenous distribution of transduced cells in the different area of the tumor. In order to avoid a bias due to sampling problem and eliminate this variability, we decided to completely dissociate the tumors and count the cells with a fluorescence microscope. This analysis showed that a small percentage of double labeled cells ranging from 0.3% to 3%, was present also in tumors that appeared exclusively DsRed-positive. The fraction of Btg2-positive cells in the tumors was dramatically reduced compared to that at the time of transplant in all tumors analyzed (Figure 2E). In contrast, in control tumors, the percentages of EGFP-positive cells were almost unchanged (Figure 2E). These results show that Btg2 expression decrease the tumorigenic potential of PDGF-induced glioma. The latency of secondary tumors generated from mixed cultures containing Btg2-transduced cells was identical to that of secondary tumor generated from control cultures (Figure S3D). This result is explained by our previous observation that the latency of secondary tumors is scarcely correlated with the number of transplanted tumorigenic cells in the range between 5000 and 50,000 cells (data not shown) and by the fact that in mixed culture containing Btg2-transduced cells is present a large fraction of tumorigenic cells not transduced with Btg2 responsible for the generation of the secondary tumor.

We then asked if the overexpression of Btg2 reduces tumorigenicity of PDGF-B cells by inducing a withdrawal from cell cycle, by increasing apoptosis or rather by slowing their cell cycle. We therefore monitored independent cultures of fully progressed PDGF-B oligodendroglioma cells transduced with Btg2-EGFP or control vector at different passages. The analysis showed that the percentage of Btg2-transduced cells decreases passage after passage (Figure 2F) and that Btg2-transduced cells express a tenfold-reduced level of Cyclin D1 mRNA as compared to the untransduced cells (Figure 2G), consistently with the known effect of Btg2 on Cyclin D1 promoter [11]. However, the fraction of proliferating cells did not change, as shown by the percentage of cells immunopositive for the marker Ki67 that was virtually identical in Btg2-transduced and control cells (86% ± 7% and 82% ± 2% respectively, Figure 2H–J), and by the intensity of immunoreactivity to Ki67 that was also unchanged (Figure 2K). No difference was found in the frequency of picnotic nuclei of transduced cells, which was 1.5% in Btg2 and 2% in control cells (data not shown), indicating that the percentage of apoptotic cells was not altered by Btg2 transfection. Taken together, these observations suggest that the overexpression of Btg2 causes an increase of cell cycle length rather that an exit from cell cycle. The reduction of Cyclin D1, which level is directly correlated with the cell cycle speed [15,16], would be compatible with this view.

Altogether these results show that the expression of Btg2 is compatible with the survival of PDGF-B overexpressing cells, although it severely limits their ability to proliferate. In this condition, it is conceivable that the progression towards fully blown malignancy provides the loss of Btg2 expression, and that those cells that have the chance to downregulate its expression rapidly become prominent in the tumor masses.

**Figure 3:**
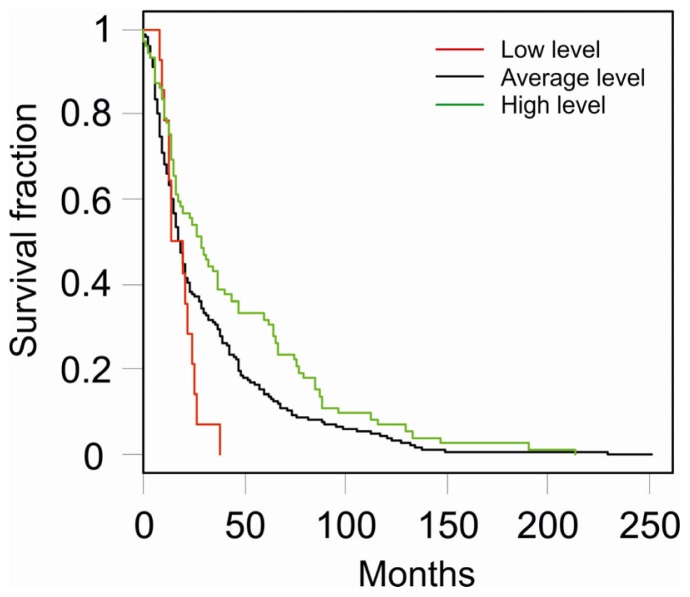
Kaplan-Meier survival analysis of patients with low-(red line), average-(black line) and high-(green line) Btg2 expression level. The groups were defined basing on the lowest geometric mean intensity of the Affymetrix probeset 201235_s_at. To the “low level” group were assigned patients showing levels two times smaller than average (*n* = 14), conversely, to the “high level” group were assigned patients showing levels two times higher than average (*n* = 72). The remaining patients were assigned to the “Average level” group (*n* = 257).

Interestingly a survival analysis performed on NCI REMBRANDT public dataset [17], showed that glioma patients displaying a Btg2 expression level twofold lower than the average have a significantly worse prognosis that those displaying a high level of Btg2 expression (median survival of 16.3 months *versus* 28.5 months respectively, rank test: *p* < 0.01, Figure 3). This result suggests that the role played by Btg2 in glioma progression is not limited to PDGF-induced oligodendrogliomas but, importantly, it extends also to human pathology.

## 3. Experimental Section

### 3.1. Animal Procedures

Animal handling conformed to the current Italian regulations about protection of the used for scientific purposes (D.lvo 27/01/1992, n. 116). Our procedures were also approved by the Ethical Committee for animal experimentation (CSEA) of the National Institute of Cancer Research and by the Italian Ministry of Health. Virus injections were performed in the ventricles of E14 mice since at a stage such that the majority of progenitor cells are still accessible from the ventricular surface and the survival of embryos after injection is higher than at earlier stages. After laparotomy in anaesthetized pregnant females, retroviral vectors were injected intrauterus in the embryonic telencephalic vesicles with a glass syringe. Reabsorbable suture was used before awakening the animals. After birth, pups were then monitored daily and killed as soon as they showed symptoms as hyper-reactivity, imbalanced stance and gait or paralysis. Brains were analyzed under a fluorescent microscope to locate the tumor which was explanted for further analysis.

Transplant of dissociated tumor cells were performed on anaesthetized adult mice using a stereotaxic apparatus and a Hamilton Syringe as described in [18]. We injected 30,000 tumor cells using the following coordinates referred to the bregma: 1 mm anterior, 1.5 mm left, 2.5 mm below. Animals were then monitored daily and killed at the first sign of neurological symptoms.

### 3.2. Retroviral Vectors

PDGF-B/GFP and PDGF-B/DsRed retroviral vectors were previously described [2,19]. The coding sequence of mouse Btg2 was inserted in the pCAG:GFP retroviral vector (kindly provided by Goetz, M., Helmholtz Center, Munich, Germany), upstream of the IRES-GFP sequence. The same pCAG:GFP vector coding only for GFP was used as control vector. The sequences of the artificial microRNAs against Btg2 were obtained using the BLOCK.iT RNAi Designer (Life Technologies) and they were cloned into the pcDNA6.2-GW/EmGFP-miR (Life Technologies). The EmGFP-miR cassette was then inserted in PDGF-B/GFP, substituting the original EGFP cassette. Details on cloning procedure are available on request. Retroviral supernatants were obtained by transiently transfecting the above-described vectors into Phoenix packaging cells and harvesting the supernatant 2 days later. The supernatants were concentrated by centrifugation and stored at −80 °C before use.

### 3.3. Cell Cultures and Immunostainings

Tumors were dissociated and sorted as described in [13]. Cultured cells were maintained in DMEM-F12 added with B27 supplement, human bFGF and EGF (10 ng/mL, Peprotech) and plated on Matrigel (1:200, BD Biosciences).

Immunostainings were done on cells fixed in 4% paraformaldehyde. Primary antibodies: rabbit polyclonal antibody against olig2 (1:500; Chemicon Temecula, CA, USA), rabbit polyclonal antibody against Ng2 (1:500; Chemicon, Temecula, CA, USA), mouse monoclonal antibody against GFAP (1:200; Sigma, St. Louis, MO, USA), rat polyclonal antibody against Ki67 (1:25; Dako, Carpinteria, CA, USA); chicken polyclonal antiserum against GFP (1:500, Abcam, Cambridge, MA, USA). Secondary antibodies: anti-mouse Dylight 549-conjugated antibody, anti-rabbit Dylight 549-conjugated antibody, anti-chicken Alexa488-conjugated antibody (1:500, Immucor, Atlanta, GA, USA), anti-rat Cy3-conjugated antibody (1:50, Dako, Carpinteria, CA, USA). Nuclei were stained via 5 min incubation in Hoechst 33342 (1 μg/mL, Sigma, St. Louis, MO, USA). Immunostainings were analyzed automatically on 16 bit microphotographs, obtained with an AXIOCAM MRM (Zeiss, Oberkochen, Germany), using an ImageJ plugin (available on request). ImageJ [14].

### 3.4. Microarray and Real-Time PCR

For microarray, RNA was extracted from sorted cells with TRIzol reagent (Invitrogen, Carlsbad, CA, USA), according to the manufacturer guidelines as described elsewhere [20]. Hybridization and scanning of Affymetrix MoGene-1.0-st chips were performed by Genopolis consortium (Milano, Italy). Data analysis was performed with Bioconductor 2.4 [21]. The Robust Multichip Average (RMA) method was then employed to calculate probe set intensity [22].

For real-time PCR, RNA was extracted with QIAzol reagent (Qiagen, Germany), following the manufacturer guidelines. cDNA was obtained from 500ng of RNA using the iScript (Bio-Rad Laboratories) retrotranscription kit. Real-time PCR was performed with iQ SyBr green supermix (Bio-Rad Laboratories). mRNA quantifications were normalized to the housekeeping gene Rpl41 (NM_018860). Sequences of the primers used are available on request.

### 3.5. Western Blot

HEK293 were cotransfected with myc-tagged Btg2 and PDGF-mirBtg2 or pCAG:GFP control vector and were harvested 3 days after transfection with a lysis buffer containing 50 mM HEPES (pH 7.5), 5 mM EDTA, 150 mM NaCl, 1% Triton^®^ X-100 detergent and protease inhibitors (Complete, Roche Applied Science). Western blot was performed as described in [23]. The level of myc-tagged protein was normalized to beta-actin level. Primary antibodies: mouse monoclonal anti-beta-actin (Sigma, St. Louis, MO, USA), mouse monoclonal anti-myc (Sigma, St. Louis, MO, USA). Secondary antibodies: anti-mouse HRP conjugated (Sigma, St. Louis, MO, USA).

## 4. Conclusions

Our previous studies demonstrated that PDGF-induced oligodendrogliomas undergo tumor progression from low to high grade, and only the latter display full blown malignant characteristics, being able to generate secondary tumors after transplant.

Here we show that the putative tumor suppressor gene Btg2 is consistently downregulated in high grade gliomas and that its downregulation, although not sufficient to allow the tumor progression, is necessary for a cell to maintain the malignant phenotype.

Interestingly, the role of Btg2 is not limited to murine PDGF-induced oligodendroglioma model but rather it seems to be extended also to human gliomas. This is suggested by our meta-analysis on public datasets showing that patients with a lower level of Btg2 have a dramatically worse prognosis than those with a higher Btg2 level.

These results encourage further analysis aimed to identify the mechanism underlying the effects of Btg2 on glioma progression. In particular it will be interesting to ascertain whether Btg2 overexpression is correlated to a decrease of glioma initiating cells, or to a switch in mode of cell division, from symmetric to asymmetric, reminiscent of that induced by Btg2 during neurogenesis.

## Supplementary Materials



## Figures and Tables

**Table 1:** Gene expression level of TP 53 and its known downstream targets.

Symb	L1	L2	H1	H1	*p*
Btg2	9.48	9.54	8.67	8.26	0.037
Trpm2	7.05	6.78	5.80	6.11	0.042
Ddb2	7.60	7.93	7.00	6.86	0.045
Vdr	5.55	5.51	5.89	6.12	>0.05
Igfbp3	10.46	10.40	10.89	11.27	>0.05
Pcna	11.82	11.51	11.98	12.09	>0.05
Cdkn1a	10.91	9.89	9.03	9.36	>0.05
Sh2d1a	4.71	4.63	4.85	4.74	>0.05
Tyrp1	5.01	4.90	4.95	5.20	>0.05
Hras1	8.77	8.65	8.70	8.81	>0.05
Mdm2	10.05	9.74	9.81	10.03	>0.05
Gml	5.00	5.37	5.39	4.96	>0.05
Trp53	9.47	9.53	8.45	10.40	>0.05

**Table 2:** Latency of gliomas induced by PDGF-mirBtg2 transduction and number of secondary tumors generated by the injection of each primary tumor in three adult animals.

Animal	Latency (days)	Secondary tumors
627B	51	0
627C	51	0
627D	52	0
630A	52	0
627E	55	0
645A	71	0
645B	71	0
630D	72	0
630E	84	0
627F	85	0
627G	105	1
646C	257	3
