# Supplementary material for: Role of Btg2 in the Progression of a PDGF-Induced Oligodendroglioma Model
Source: Int J Mol Sci. 2012 Nov 12;13(11):14667–78. doi: 10.3390/ijms131114667 (PMC3509603; doi:10.3390/ijms131114667)
Supplement: Supplementary file 1 [file ijms-13-14667-s001.pdf]

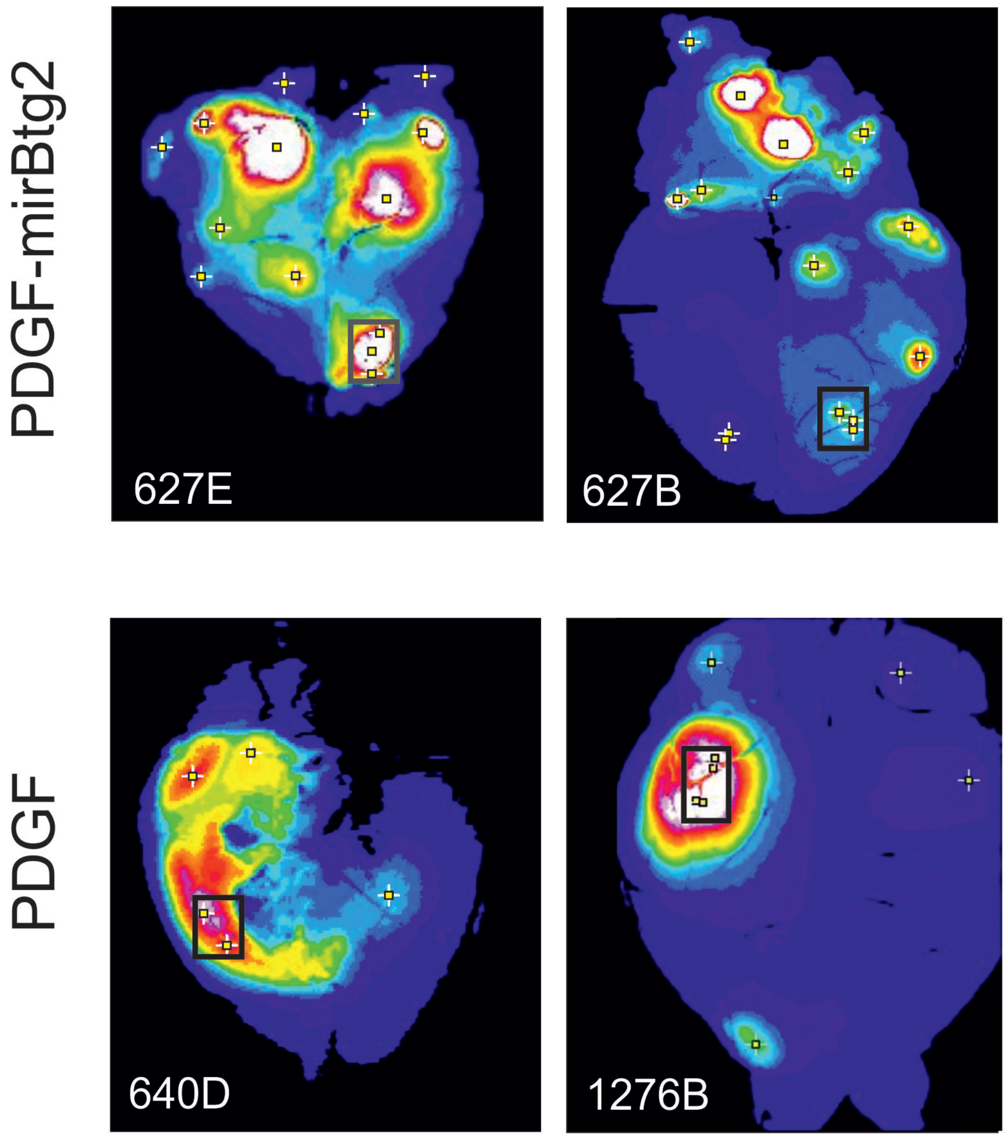

PDGF-mirBtg2

mouse	nr. of foci
627A	11
627B	12
627D	7
627C	12
627E	12
627F	3
627G	3
630A	18
average	*10*

PDGF

mouse	nr of foci
632B	2
634C	3
634D	2
640D	4
1204A	3
1275A	8
1276A	2
1276B	5
average	*3.6*

Suppl. figure 1. Example of pictures analysed to sample the number of foci in the respective brains. Pictures where analysed with ImageJ (W.S. Rasband, ImageJ, US National Institutes of Health, Bethesda, MD; http://rsb.info.nih.gov/ij/, 1997-2012) using the plugin "find maxima". Maxima are depicted onto false color images showing the level of EGFP. Maxima within the same colored area were treated as a single focus (for example those included in black rectangles in the pictures).
Tables summarize the number of foci identified in each picture.

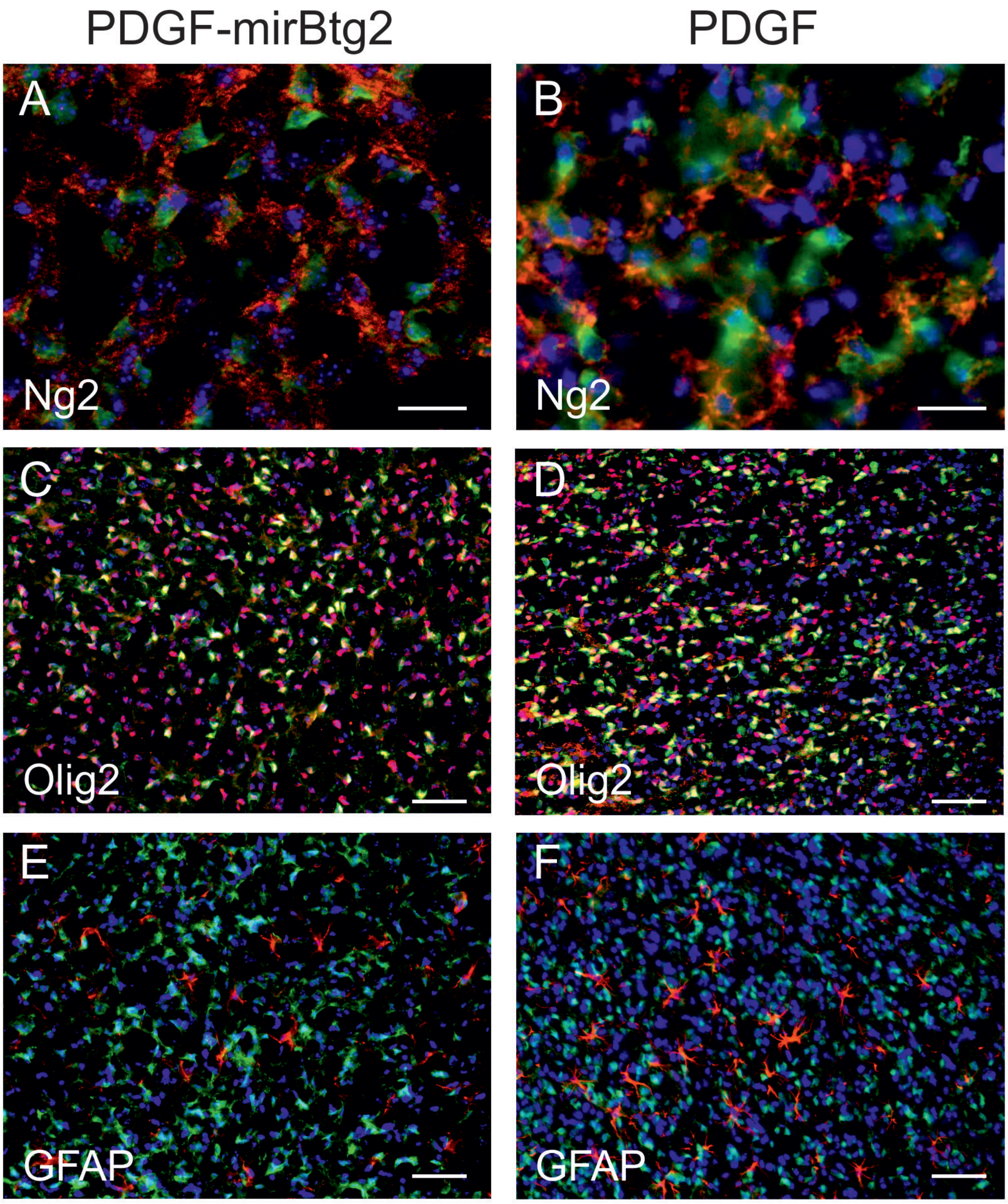

Suppl. Figure 2. Micrographs of sections of brains injected at E14 with a PDGF-mirBtg2 or a PDGF expressing retrovirus, showing the immunoreactivity for the indicated markers in red, EGFP in green, and Hoechst 33342 for nuclear staining in blue. Scale bars: 20 µm (A-B) 50 µm (C-F).

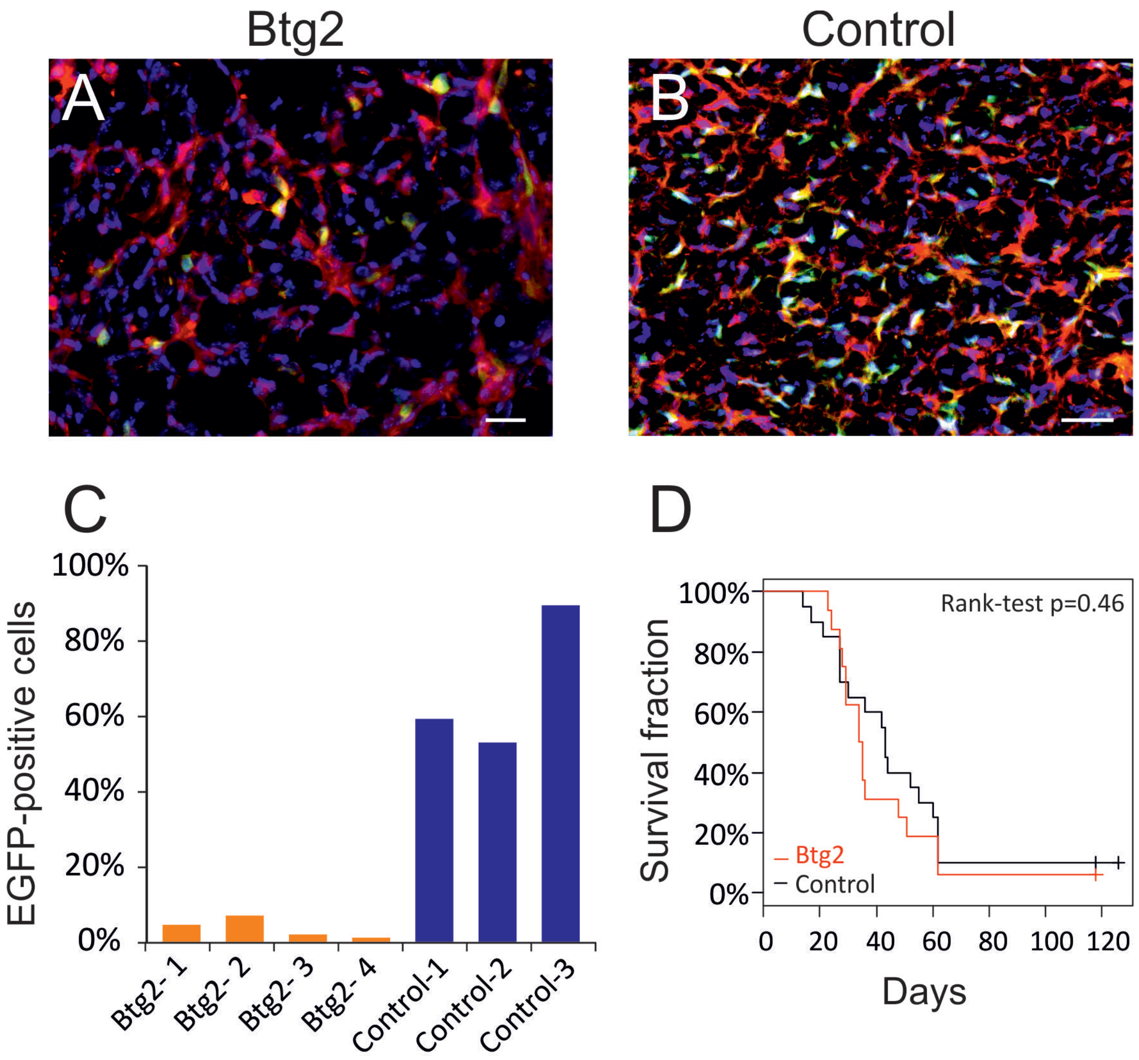

Suppl. Figure 3. (A-B) Micrographs of sections of brains injected with Btg2- (A) or control- (B) transduced glioma cells. PDGF-expressing cells are shown in red, Btg2- or EGFP-expressing cells in green and nuclei in blue. (C) Quantification of the percentage of EGFP-immunopositive cells in tumors generated by PDGF-induced gliomas cells transduced with Btg2-expressing or control retroviral vectors. (D) Kaplan-Meier plot of animals injected with PDGF-induced gliomas cells transduced with Btg2-expressing or control retroviral vectors. Scale bars: 20 μm.
